# Remimazolam versus propofol for elderly patients undergoing laparoscopic gastrointestinal surgery: a randomized trial concentrating on delirium and hemodynamic stability

**DOI:** 10.3389/fmed.2026.1992969

**Published:** 2026-10-16

**Authors:** Hongna Fan, Xiaotong Chen, Linlin Xu, Bin Zou, Da Li, Cheng Zhang, Jianyu Zu, Yugang Diao

**Affiliations:** 1 Department of Anesthesiology, General Hospital of Northern Theater Command, Shenyang, China; 2 Key Laboratory of Perioperative Critical Care Medicine of Liaoning Province, Shenyang, China; 3 Shenyang Clinical Medical Research Center for Anesthesiology and Perioperative Medicine, Shenyang, China; 4 Postgraduate Training Base, General Hospital of Northern Theater Command, Dalian Medical University, Dalian, China; 5 Department of General Surgery, General Hospital of Northern Theater Command, Shenyang, China; 6 Department of Anesthesiology, Suzhou BOE Hospital, Suzhou, China

**Keywords:** elderly patients, gastric and colorectal surgery, postoperative delirium, propofol, remimazolam

## Abstract

**Background:**

Postoperative delirium (POD) is a common complication in elderly surgical patients that can impede recovery. While remimazolam offers superior hemodynamic stability compared to propofol, its impact on POD incidence remains unclear. This randomized trial aimed to determine whether two induction doses of remimazolam are more effective than propofol in reducing POD and improving hemodynamic stability in older patients undergoing laparoscopic gastrointestinal surgery.

**Methods:**

In this prospective trial, 186 patients (aged 60–80 years) were randomized to receive anesthesia induction with remimazolam 0.15 mg/kg (R1), remimazolam 0.3 mg/kg (R2), or propofol 1.5 mg/kg (P). The primary endpoint was the incidence of POD within 5 days post-surgery, assessed using the 3-Minute Diagnostic Interview for Confusion Assessment Method. Secondary endpoints included hemodynamic parameters, recovery times, and adverse events.

**Results:**

The incidence of POD was 12.9% in the R1 group, 11.3% in the R2 group, and 6.5% in the *P* group (*P* = 0.467). Both remimazolam groups exhibited significantly higher mean arterial pressure following induction and intubation (both *P* < 0.05). Furthermore, remimazolam was associated with a lower incidence of treated hypotension (*P* = 0.010), fewer bradycardia events (*P* < 0.001), and reduced vasopressor requirements (*P* < 0.05). However, the time to loss of consciousness (*P* < 0.001) and extubation time (*P* < 0.001) were longer with remimazolam.

**Conclusions:**

Remimazolam at induction doses of 0.15 mg/kg and 0.3 mg/kg did not reduce the incidence of POD compared with propofol. Nevertheless, the study was not adequately powered to exclude a clinically relevant difference in POD incidence given the substantially lower-than-expected event rate, as no statistically significant between-group difference was detected. In addition, remimazolam at these doses was associated with higher post-induction blood pressure, fewer hypotensive events, and lower vasopressor requirements compared with propofol. Therefore, remimazolam at these induction doses may serve as suitable alternatives for this vulnerable patient population.

**Clinical trial registration:**

https://www.chictr.org.cn, Chinese Clinical Trial Registry ChiCTR2400082176.

## Introduction

Postoperative delirium (POD) remains a serious complication in elderly surgical patients, associated with prolonged hospital stays, increased healthcare costs, and higher morbidity (1–5). Despite the association of perioperative benzodiazepines with delirium (6, 7), these agents continue to be frequently utilized in clinical practice.

Remimazolam tosylate is a novel, ultra-short-acting benzodiazepine characterized by a rapid onset, quick offset, and organ-independent metabolism. Compared with propofol, remimazolam offers superior hemodynamic stability (8). Conversely, propofol frequently causes hypotension and bradycardia. In older patients, these hemodynamic fluctuations may reduce cerebral blood flow, potentially increasing the risk of POD.

Given these properties, remimazolam represents a potentially more stable alternative for anesthesia induction. However, evidence regarding its impact on POD in high-risk elderly patients undergoing major abdominal surgery remains scarce and inconsistent (9–11). Furthermore, the optimal induction dose for this specific population has not yet been established.

Therefore, we conducted this prospective, randomized, three-arm superiority trial to determine whether remimazolam (0.15 or 0.3 mg/kg) is superior to propofol in reducing the incidence of POD within 5 days following laparoscopic gastrointestinal surgery in elderly patients. Secondary objectives included comparing hemodynamic stability and perioperative adverse events across the three groups.

## Methods

### Ethical approval and study registration

This study was conducted in accordance with the principles of the Declaration of Helsinki and received approval from the Ethics Committee of Northern Theater General Hospital (Approval No. Y[2023]164). The trial was registered with the Chinese Clinical Trial Registry (Registration ID: ChiCTR2400082176; Registration Date: March 22, 2024). Written informed consent was obtained from all participants or their legal representatives prior to enrollment. The study design and reporting adhered to the Consolidated Standards of Reporting Trials (CONSORT) guidelines.

This prospective, randomized, single-center, three-arm superiority trial was designed to evaluate whether remimazolam, administered at two different induction doses, is superior to propofol in reducing the incidence of postoperative delirium (POD).

### Patient selection, randomization, and blinding

Between March and November 2024, we assessed consecutive patients aged 60–80 years scheduled for elective laparoscopic gastrointestinal surgery at Northern Theater General Hospital. Eligible participants met the following inclusion criteria: American Society of Anesthesiologists (ASA) physical status II–III and a body mass index (BMI) of 18–30 kg/m^2^. We excluded patients with preoperative delirium or dementia (assessed using the education-adjusted Mini-Mental State Examination; Supplementary Table S1), communication barriers or severe sensory impairments, diagnosed psychiatric disorders, known hypersensitivity to benzodiazepines, or an anticipated difficult airway. Therefore, the findings of this study apply only to elderly patients without preexisting cognitive impairment, and caution should be exercised when extrapolating these results to those with pre-operative cognitive dysfunction.

Eligible patients were randomly assigned in a 1:1:1 ratio to one of three parallel groups: remimazolam 0.15 mg/kg (R1 group), remimazolam 0.3 mg/kg (R2 group), or propofol 1.5 mg/kg (*P* group, active control). Randomization was performed using a computer-generated block randomization sequence (block size of 6) with a fixed seed (123456) via SPSS 25.0 software (IBM Corp., Armonk, NY, USA). An independent statistician, who was not involved in patient recruitment or clinical care, managed the concealed allocation sequence. Upon submission of baseline data by investigators, a centralized randomization system automatically assigned participants to their respective groups.

Both participants and outcome assessors remained blinded to group assignments throughout the trial. Due to the distinct physical properties and dosing requirements of the study drugs, the attending anesthesiologist administering the anesthesia was necessarily unblinded. However, this anesthesiologist was strictly excluded from all postoperative outcome assessments, trial design, and data analysis to maintain the integrity of the blinding.

### Perioperative protocol

#### Preoperative preparation

Patients followed standard fasting guidelines (8 h for solids, 2 h for clear fluids). All patients received oral polyethylene glycol electrolyte powder (III) at 8:00 PM on the day before surgery, along with 1000 mL of sodium chloride and glucose injection. No preoperative benzodiazepines were administered. Antihypertensive medications were continued until the morning of surgery.

#### Intraoperative management

Upon arrival in the operating room, standard monitoring was initiated, including electrocardiography, heart rate (HR), invasive radial arterial blood pressure (BP) monitoring, bispectral index (BIS), pulse oximetry (SpO₂), and end-tidal carbon dioxide (ETCO₂). After a 10-min stabilization period, baseline parameters were recorded. Preoxygenation was performed via face mask (oxygen flow 5 L/min) for 3 min before anesthesia induction.

#### Anesthesia induction and maintenance in the three study arms

The induction doses of all sedative agents were administered via a slow intravenous bolus over a duration of 60 s.

R1 Group (Low-dose Remimazolam): Induction was performed with remimazolam (0.15 mg/kg), sufentanil (0.3 µg/kg), and rocuronium (0.6 mg/kg). In case of no loss of consciousness (LOC) within 3 min, a rescue dose of remimazolam (0.1 mg/kg) was administered over 2 min. Anesthesia was maintained with remimazolam infusion at the dose of 1–1.5 mg/kg/h.

R2 Group (High-dose Remimazolam): Induction and maintenance protocols were identical to the R1 group, except for the use of a higher induction dose of remimazolam (0.3 mg/kg), sufentanil (0.3 µg/kg), and rocuronium (0.6 mg/kg).

*P* Group (Propofol Control): Induction was performed with propofol (1.5 mg/kg), sufentanil (0.3 µg/kg), and rocuronium (0.6 mg/kg), with a rescue dose of 0.5 mg/kg administered if LOC was not achieved within 3 min. Anesthesia was maintained with propofol infusion at the dose of 4–12 mg/kg/h.

Loss of consciousness (LOC) was defined as the simultaneous loss of both verbal response (no response to a loud verbal command, such as ‘Open your eyes!’) and loss of eyelash reflex. This was assessed by an anesthesiologist who was blinded to the group assignment.

Following tracheal intubation, all three groups received a remifentanil infusion at 0.05–2 μg/kg/min for maintenance of intraoperative analgesia. Tracheal intubation was completed within 5 min after induction. Additional doses of rocuronium (0.15 mg/kg) were administered to maintain neuromuscular blockade, guided by qualitative neuromuscular monitoring (train-of-four count) and clinical assessment of surgical conditions. Ventilator settings were standardized as follows: FiO₂ of 50%; tidal volume of 6 mL/kg, respiratory rate of 12 breaths/min, and PEEP of 5 cm H₂O. The Bispectral Index(BIS) was maintained between 40 and 60, and blood pressure(BP) was kept within 20% of baseline values.

#### Management of hemodynamic instability

During anesthesia induction and the immediate post-induction period, hemodynamic instability was managed with intravenous boluses: hypotension (systolic BP decrease >20% from baseline or <90 mmHg) was treated with phenylephrine 100 μg; hypertension (systolic BP increase >20% from baseline) with urapidil 5 mg; bradycardia (HR ≤ 50 bpm) with atropine 0.5 mg; and tachycardia (HR ≥ 100 bpm) with esmolol 20 mg.

For the maintenance phase of anesthesia, the management protocol was as follows: For sustained hypotension requiring repeated phenylephrine boluses, a continuous infusion of norepinephrine was initiated and titrated according to the protocol to maintain BP within 20% of baseline values. Hypertension, bradycardia, and tachycardia during maintenance were managed with the same bolus agents (urapidil, atropine, esmolol) used during induction.

Definition of Hypotension Metrics: For analysis, two hypotension-related outcomes were defined: 1) ‘post-induction hypotension’: the proportion of patients experiencing at least one episode of systolic BP decrease >20% from baseline or <90 mmHg during induction and the immediate post-induction period. 2) ‘Treated hypotension’: the proportion of patients requiring a phenylephrine bolus (100 µg) for such an episode.

#### Anesthesia recovery and postoperative analgesia

At the conclusion of surgery, all anesthetic infusions were discontinued. Neuromuscular blockade was reversed using neostigmine (0.04 mg/kg) and atropine (0.02 mg/kg). Extubation was performed only after the patient met the following standard criteria: recovery of consciousness (ROC), defined as the ability to follow commands (e.g., “Open your eyes”) and demonstrate purposeful movement, accompanied by adequate airway reflexes (cough and gag reflex); stable spontaneous ventilation, indicated by a respiratory rate＞8 breaths/min, tidal volume＞5 mL/kg, and end-tidal CO₂＜50 mmHg; and hemodynamic stability, defined as a mean arterial pressure within 20% of baseline values. A blinded anesthesiologist assessed ROC and readiness for extubation. Flumazenil was not administered in any case. Extubation time was recorded as the interval from the end of surgery to the removal of the endotracheal tube.

Patients were discharged from the post-anesthesia care unit (PACU) upon meeting the following criteria: a modified Aldrete score （score discharged from the post-anesthesia care unitre within 20% of the preoperative baseline), absence of active surgical site bleeding, and adequate pain control (Numerical Rating Scale [NRS] score≤3). The duration of the PACU stay was calculated from the time of admission to discharge.

Postoperative analgesia was administered via a patient-controlled analgesia (PCA) pump containing oxycodone (0.8 mg/kg), dexamethasone (10 mg), and ondansetron (24 mg) diluted in 100 mL of normal saline. The pump was programmed with a 2 mL loading dose, a continuous infusion rate of 2 mL/h, a 2 mL bolus dose, and a 15-minute lockout interval. This standardized regimen ensured identical weight-based opioid exposure for all patients during the first 48 postoperative hours. The PCA was maintained for 48 h, followed by intravenous administration of flurbiprofen axetil (50 mg) every 8 h. No additional opioids were administered outside of the PCA protocol. In addition to the antiemetic prophylaxis provided by the PCA regimen (dexamethasone and ondansetron), a dedicated prophylactic intravenous dose of ondansetron (8 mg) was administered approximately 30 min before the end of surgery.

### Study endpoints

The primary endpoint was the incidence of postoperative delirium (POD) within 5 days following surgery.

Secondary endpoints included the classification of POD subtypes, time of onset, and duration of symptoms. Hemodynamic parameters: heart rate(HR), systolic blood pressure (SBP), and diastolic blood pressure (DBP)—were recorded at predefined time points: T0 (baseline), T1 (immediately after induction), T2 (3 min after induction), T3 (immediately after intubation), and T4 (5 min after intubation). We also assessed the incidence of induction-related adverse events, including hypotension, bradycardia, and injection pain, as well as the time to loss of consciousness following sedative administration. Procedural metrics included the durations of surgery and anesthesia, total intraoperative consumption, and mean infusion rates of sedatives, analgesics, and vasoactive agents. Recovery quality was evaluated based on extubation time, duration of the post-anesthesia care unit (PACU) stay, and the incidence of emergence agitation. Additionally, postoperative pain severity was assessed using the Numerical Rating Scale (NRS) on postoperative days 1–3, and the incidence of postoperative nausea and vomiting (PONV) was recorded.

POD was assessed by blinded assessors twice daily (08:00–10:00 and 16:00–20:00) for 5 consecutive days using the 3-Minute Diagnostic Interview for Confusion Assessment Method (3D-CAM). Emergence agitation was evaluated using the Riker Sedation-Agitation Scale (SAS, Supplementary Table S2), with a score of ≥5 defined as agitation. Postoperative pain intensity was measured using the NRS on postoperative days 1 through 3.

### Sample size calculation and statistical analysis

#### Sample size calculation

The sample size was determined *a priori* using PASS 25.0 software (IBM Corp., Armonk, NY, USA). The anticipated incidence of postoperative delirium (POD) in the propofol control group was estimated at 33%. This conservative estimate was derived from previous studies reporting a POD incidence of up to 30% in elderly patients undergoing radical resection for colorectal cancer (12), and accounts for the high-risk profile of the study population (aged 60–80 years undergoing laparoscopic abdominal surgery) as well as the potential contribution of propofol-induced hypotension to delirium risk. We hypothesized that remimazolam would reduce the incidence of POD to 15% in the higher-dose group (R2) and to 10% in the lower-dose group (R1), consistent with a potential dose-dependent protective effect. Assuming a two-sided *α* level of 0.05, 80% statistical power (1 – *β*), and a 1:1:1 allocation ratio, a minimum of 155 participants was required. To account for an anticipated attrition rate of 20%, the final target sample size was set at 186 patients, with 62 participants allocated to each group.

#### Statistical analysis

Statistical analyses were performed using SPSS 25.0 software. As this was a three-arm superiority trial, normally distributed continuous variables were expressed as mean. Assuming a two-sided test, statistical analysis was performed using one-way analysis of variance (ANOVA). *Post-hoc* comparisons were conducted using the Bonferroni correction (when equal variances were assumed) or Tamhane's T2 test (when equal variances were not assumed). Non-normally distributed continuous variables were expressed as median (interquartile range) and analyzed using the Kruskal-Wallis H test, with Bonferroni correction applied for within-endpoint pairwise between-group comparisons. Categorical variables were expressed as number (%) and compared using the Chi-square test. For the primary outcome (POD incidence), the overall three-group comparison was performed with Pearson chi-square test. All three pairwise comparisons (R1 vs. propofol, R2 vs. propofol, and R1 vs. R2) were prespecified in the trial protocol. Bonferroni correction was applied for these three prespecified pairwise comparisons. Two-level multiplicity adjustment was applied to control type I error for secondary-outcome analyses. Bonferroni correction was used to adjust for multiple independent secondary endpoints. For post-hoc pairwise simple-effect comparisons across groups and time-points within repeated hemodynamic outcomes, the Holm-Bonferroni sequential correction was adopted. Longitudinal hemodynamic data (heart rate, systolic and diastolic blood pressure) were analyzed using generalized estimating equation (GEE) models to evaluate group-by-time interaction. The Shapiro-Wilk test and Levene's test were used to evaluate normality and homogeneity of variance, respectively. A two-tailed *P* < 0.05 was considered statistically significant.

## Results

### Patients’ characteristics

Patient enrollment, randomization, and follow-up are detailed in the CONSORT flow diagram (Figure 1). Of the 302 patients screened for eligibility, 186 were enrolled and randomly assigned to the three study groups. All 186 enrolled patients completed the study.

**Figure 1:**
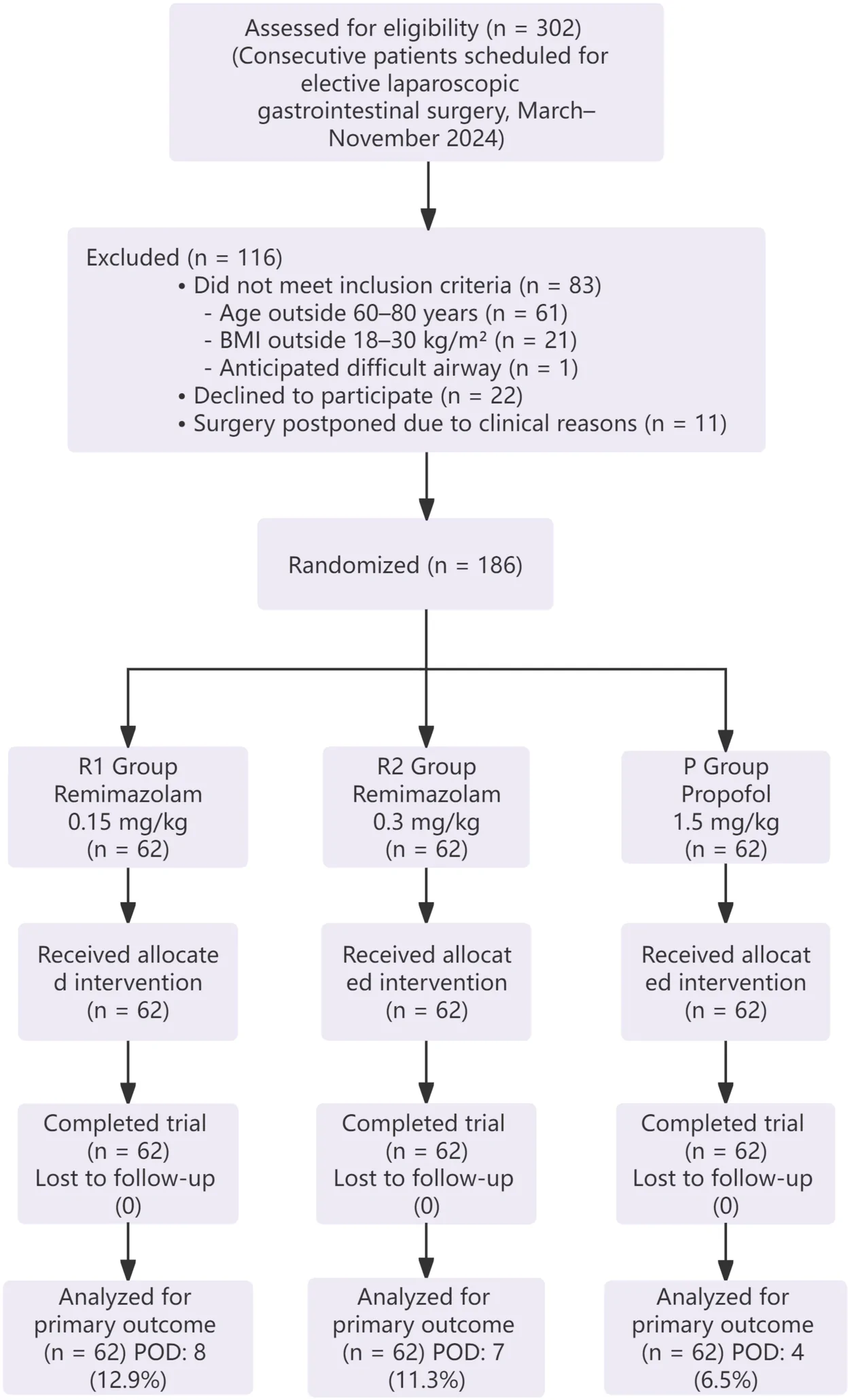
CONSORT flow diagram of patient enrollment, randomization, and follow-up. Between March and November 2024, 302 consecutive patients scheduled for elective laparoscopic gastrointestinal surgery were assessed for eligibility. After exclusions (*n* = 116), 186 patients were randomized in a 1:1:1 ratio to receive induction with remimazolam 0.15 mg/kg (R1 group, *n* = 62), remimazolam 0.3 mg/kg (R2 group, *n* = 62), or propofol 1.5 mg/kg (*P* group, *n* = 62). All patients received the allocated intervention, completed the trial, and were included in the intention-to-treat analysis. POD indicates postoperative delirium.

There were no significant differences in baseline demographic or clinical characteristics among the three groups, confirming successful randomization (Table 1). The types of surgical procedures performed were comparable across the three groups (*P* = 0.847). The most common procedures were rectal surgery (R1: 40.3%, R2: 43.5%, P: 33.9%) and gastric surgery (R1: 30.6%, R2: 27.4%, P: 30.6%). A complete breakdown of surgical procedures is provided in Supplementary Table S3.

**Table 1:** The baseline characteristics of the included patients (*n* = 186).

Demographic characteristics	R1 group (*n* = 62)	R2 group (*n* = 62)	*P* group (*n* = 62)	*P*
Age, y	69.1 ± 4.9	69.8 ± 4.6	68.0 ± 4.7	0.108
Male, *n* (%)	40 (64.5)	40 (64.5)	43 (69.4)	0.806
BMI (kg/m^2^)	23.4 (21, 26)	23.7 (22.4,26.2)	23.8 (21.7,25.5)	0.569
MMSE (score)	26 (24, 28)	27 (25, 29)	25.5 (23, 28)	0.228
ASA grade, *n* (%)				0.152
II	38 (61.3)	47 (75.8)	46 (74.2)	
III	24 (38.7)	15 (24.2)	16 (25.8)	
Comorbidity, *n* (%)				
hypertension	30 (48.4)	34 (54.8)	23 (37.1)	0.134
diabetes mellitus	3 (4.8)	5 (8.1)	5 (8.1)	0.718

BMI, body mass index; MMSE, mini-mental state examination; ASA, American Society of Anesthesiologists.

### The influence of remimazolam on incidence of postoperative delirium

In this superiority trial, remimazolam did not demonstrate superiority to propofol in reducing the incidence of POD. The incidence rates were 12.9% (8/62) in the R1 group, 11.3% (7/62) in the R2 group, and 6.5% (4/62) in the *P* group (*P* = 0.467). An exploratory analysis revealed that the risk differences compared with propofol were 6.4% (95% CI: −3.8% to 16.7%) for R1 and 4.8% (95% CI: −5.1% to 14.8%) for R2.The corresponding relative risks were 2.00 (95% CI: 0.64 to 6.30) for R1 versus propofol and 1.75 (95% CI: 0.54 to 5.68) for R2 versus propofol. The time to onset, duration, and subtypes of delirium were also comparable across the three groups (all *P* > 0.05, Table 2). Of note, the observed POD incidence was far lower than anticipated, which limited our ability to rule out a clinically meaningful between-group difference.

**Table 2:** The POD incidence in the included patients.

Variables	R1 group (*n* = 62)	R2 group (*n* = 62)	*P* group (*n* = 62)	*P*
Incidence of POD, *n* (%)	8 (12.9)	7 (11.3)	4 (6.5)	0.467
The time of POD occurred, d	1 (1, 2)	2 (1, 2)	1 (1, 1.8)	0.567
The duration of POD, d	1 (1, 1.8)	1 (1, 1)	1 (1, 1)	0.233
Type of delirium, *n* (%)^a^				0.722
hypoactive	6 (75)	4 (57.1)	3 (75)	
hyperactive	2 (25)	3 (42.9)	1 (25)	
Mixed	0 (0)	0 (0)	0 (0)	
POD (95%CI)	4–21%	3–19%	0–13%	

POD, postoperative delirium.

a data were analyzed among patients with POD.

### Hemodynamic outcomes

In contrast to the primary outcome, both remimazolam groups demonstrated markedly superior intraoperative hemodynamic outcomes compared with the propofol group. At most time points post-induction (T1-T4), HR, SBP, and DBP were consistently and significantly higher in the R1 and R2 groups than those in the *P* group (e.g., SBP at T2: R1 119.4 ± 16.5 mmHg, R2 118.7 ± 11.0 mmHg vs. *P* 108.4 ± 11.7 mmHg, *P* < 0.001; Tables 3–5).

**Table 3:** Comparison of heart rate at different time points among the three groups.

Time points	R1 group (*n* = 62)	R2 group (*n* = 62)	*P* group (*n* = 62)	*P*
T0	72.2 ± 12.4	72.7 ± 10.7	73.7 ± 11.9	0.765
T1	71.8 ± 12.7^&^	72.1 ± 10.7^&^	65.7 ± 10.8^#^	0.002
T2	69.2 ± 10.5^#&^	69.7 ± 11.4^#&^	62.1 ± 12.0^#^	<0.001
T3	70.7 ± 11.0^&^	70.6 ± 10.7^&^	66.0 ± 9.6^#^	0.019
T4	72.0 ± 12.8^&^	71.1 ± 10.2^&^	66.4 ± 10.0^#^	0.008

Data were presented as mean ± SD (beat per min); T0: Baseline; T1: Immediately after induction; T2: 3 min after induction; T3: Immediately after intubation; T4: 5 min after intubation.

Post-hoc inter-group comparisons (R1/R2 vs P) were adjusted using Holm-Bonferroni sequential correction. Notably, the statistical significance of these pairwise comparisons remained unchanged after multiplicity correction. Symbol “&” indicates significant differences after correction, not unadjusted *P*-values. Group-by-time interaction was assessed using generalized estimating equation (GEE), with full effect estimates and 95% CIs presented in Supplementary Table S4-5.

# Compared with T0 in the same group (*P* < 0.05).

& Compared with *P* group (*P* < 0.05).

**Table 4:** Comparison of systolic blood pressure at different time points among the three groups.

Time points	R1 group (*n* = 62)	R2 group (*n* = 62)	*P* group (*n* = 62)	*P*
T0	152.4 ± 19.7	152.3 ± 19.0	148.5 ± 18.3	0.423
T1	135.3 ± 23.1^#&^	129.9 ± 20.3^#&^	120.5 ± 20.3^#^	0.001
T2	119.4 ± 16.5^#&^	118.7 ± 11.0^#&^	108.4 ± 11.7^#^	<0.001
T3	123.6 ± 19.7^#&^	122.8 ± 18.7^#&^	113.2 ± 9.5^#^	<0.001
T4	125.6 ± 20.2^#&^	125.9 ± 16.2^#&^	116.8 ± 15.0^#^	0.002

Data were presented as mean ± SD (mmHg). *Post-hoc* inter-group comparisons (R1/R2 vs P) were adjusted using Holm-Bonferroni sequential correction. Notably, the statistical significance of these pairwise comparisons remained unchanged after multiplicity correction. Symbol “&” indicates significant differences after correction, not unadjusted *P*-values. Group-by-time interaction was assessed using generalized estimating equation (GEE), with full effect estimates and 95% CIs presented in Supplementary Table S6-7.

# Compared with T0 in the same group (*P* < 0.05).

& Compared with *P* group (*P* < 0.05).

**Table 5:** Comparison of diastolic blood pressure at different time points among the three groups.

Time points	R1 group (*n* = 62)	R2 group (*n* = 62)	*P* group (*n* = 62)	*P*
T0	76.0 ± 11.4	74.2 ± 11.0	75.5 ± 9.9	0.624
T1	67.9 ± 10.2^#&^	66.1 ± 10.2^#&^	62.2 ± 6.2^#^	<0.001
T2	61.5 ± 10.8^#&^	60.4 ± 10.6^#&^	53.7 ± 5.8^#^	<0.001
T3	62.7 ± 12.1^#&^	62.1 ± 12.5^#&^	56.6 ± 4.9^#^	<0.001
T4	66.5 ± 10.4^#&^	65.9 ± 9.0^#&^	60.5 ± 4.3^#^	<0.001

Data were presented as mean ± SD (mmHg). *Post-hoc* inter-group comparisons (R1/R2 vs P) were adjusted using Holm-Bonferroni sequential correction. Notably, the statistical significance of these pairwise comparisons remained unchanged after multiplicity correction. Symbol “&” indicates significant differences after correction, not unadjusted *P*-values. Group-by-time interaction was assessed using generalized estimating equation (GEE), with full effect estimates and 95% CIs presented in Supplementary Table S8-9.

# Compared with T0 in the same group (*P* < 0.05).

& Compared with *P* group (*P* < 0.05).

### Secondary and safety outcomes

Induction and Recovery: The time to loss of consciousness was significantly longer in the remimazolam groups (R1: 30.2 s, R2: 28.4 s) than in the propofol group (24.7 s). Extubation time and PACU stay duration were also significantly prolonged in the R1 and R2 groups (*P* < 0.05 for all; Table 6). Vasoactive Drug Use: The requirements for phenylephrine and norepinephrine were significantly lower in the R1 and R2 groups than in the *P* group during both induction and maintenance of anesthesia (*P* < 0.05 for all; Table 6). Adverse Events: Consistent with the observed hemodynamic benefits, adverse events related to cardiovascular instability were significantly less frequent in the remimazolam groups. Unlike the propofol group, where the incidence of injection pain was 22.6%, no patients in either remimazolam group reported injection pain (*P* < 0.001). The requirement for vasopressor treatment for hypotension was significantly lower in the R1 and R2 groups (12.9% and 14.5%, respectively) than in the propofol group (30.6%; *P* = 0.010). Furthermore, the incidence of bradycardia and hypotension following induction was lower in both remimazolam groups compared with the propofol group (*P* < 0.001).

**Table 6:** Comparison of other perioperative indicators among the three groups.

Variables	R1 group (*n* = 62)	R2 group (*n* = 62)	*P* group (*n* = 62)	*P*
LOC (s)	30.2 ± 5.0^&^	28.4 ± 5.0^&^	24.7 ± 4.4	<0.001
Operation duration (H), median (IQR)	3.0 (2.7–3.7)	3.0 (2–3.7)	2.8 (2.2–3.5)	0.094
Anesthesia duration (H), median (IQR)	3.3 (2.8–3.9)	3.1 (2.2–4)	3.0 (2.4–3.9)	0.348
Total dosage of sedatives (mg)	175.7 ± 61.3	176.0 ± 72.7	770.2 ± 306.9	
Average infusion rate of sedatives (mg/kg/h), median (IQR)	0.8 (0.7–1.0)	0.9 (0.8–1.0)	4.0 (3.6–4.1)	
Total dosage of remifentanil (mg)	5.0 ± 1.6	5.0 ± 2.3	4.3 ± 1.7	0.056
Average infusion rate of remifentanil (µg/kg/h)	0.4 ± 0.1	0.4 ± 0.3	0.4 ± 0.1	0.350
Total dosage of phenylephrine used (µg), median (IQR)	0.0 (0.0–0.0)^&^	0.0 (0.0–0.0)^&^	0.0 (0.0–100.0)	0.003
Total dosage of noradrenaline (mg), median (IQR)	0.2 (0.0–0.5)^&^	0.3 (0.0–0.5)^&^	0.7 (0.4–1.3)	<0.001
Average infusion rate of noradrenaline (ug/kg/min), m (IQR)	1.1 (0.0–3.0)^&^	1.3 (0.0–2.5)^&^	4.0 (2.7–6.6)	<0.001
Recovery Quality				
Incidence of restlessness during the awakening period, *n* (%)	4 (6.5)	5 (8.1)	2 (3.2)	0.508
Extubation time (min), median (IQR)	32.5 (23.5–45.5)^&^	30.0 (22.0–41.3)^&^	17.5 (13.0–25.3)	<0.001
PACU stay duration (min), median (IQR)	53.5 (42.0–68.0)^&^	52.5 (40.0–65.8)^&^	45.0 (40.0–53.0)	0.008
Pain				
First day after surgery (score), median (IQR)	2.5 (1.0–3.0)	2.0 (0.75–4.0)	2.0 (1.0–3.0)	0.762
Second day after surgery (score), median (IQR)	1.0 (0.0–2.0)	1.0 (0.0–2.0)	0.0 (0.0–1.25)	0.398
Third day after surgery (score), median (IQR)	0.0 (0.0–0.0)	0.0 (0.0–0.0)	0.0 (0.0–0.0)	0.880
Postoperative opioid consumption (mg MME)	77 (70–85)	77 (70–85)	77 (70–85)	>0.99

IQR, Interquartile range; LOC, Loss of consciousness.

& Compared with *P* group (*P* < 0.05).

Other Outcomes: There were no significant differences among the three groups in the incidence of emergence agitation, postoperative pain scores, postoperative nausea and vomiting (PONV), or postoperative opioid consumption (all *P* > 0.05; Tables 6, 7).

**Table 7:** Comparison of post-induction adverse events among the three groups.

Variables	R1group (*n* = 62)	R2group (*n* = 62)	*P* group (*n* = 62)	*P*
Injection Pain (%)	0 (0)^&^	0 (0)^&^	14 (22.6)	<0.001
Post-Induction Hypotension, *n* (%)*	47 (75.8)^&^	49 (79.0)^&^	57 (91.9)	0.045
Treated hypotension, *n* (%)	8 (12.9)	9 (14.5)	19 (30.6)	0.010
Post-Induction bradycardia, *n* (%)	3 (4.8)^&^	4 (6.5)^&^	16 (25.8)	<0.001
Postoperative nausea and vomiting, *n* (%)	23 (37.1)	22 (35.5)	19 (30.6)	0.752

Treated hypotension: Defined as the proportion of patients requiring a phenylephrine bolus (100 µg) for an episode of hypotension (SBP decrease >20% from baseline or <90 mmHg) during induction and the immediate post-induction period.

Injection pain: Patient-reported pain during intravenous administration of sedative agents.

Bradycardia: Heart rate (HR) < 50 beats per minute (bpm).

& Compared with *P* group (*P* < 0.05).

*Post-Induction Hypotension: Defined as the proportion of patients experiencing at least one episode of systolic blood pressure (SBP) decrease >20% from baseline or <90 mmHg during induction and the immediate post-induction period.

## Discussion

The principal finding of this superiority-designed trial is that remimazolam did not demonstrate superiority over propofol in reducing the incidence of postoperative delirium (POD), despite providing superior hemodynamic outcomes. This finding is clinically relevant given that POD affects up to 25.4% of elderly patients undergoing gastrointestinal surgery (9–11). Although perioperative benzodiazepine use has been identified as a risk factor for delirium (13), emerging evidence indicates that the impact on delirium varies according to pharmacokinetics. Long-acting benzodiazepines have been more strongly associated with POD than short-acting agents (14), potentially owing to drug accumulation in elderly patients with diminished organ function (15). Remimazolam, an ultra-short-acting benzodiazepine metabolized rapidly into an inactive metabolite (16), possesses pharmacological advantages, including reduced cortisol levels, which may provide neuroprotective benefits (17, 18).

Consistent with recent studies (12, 19–23), the present study showed numerically similar POD incidence between agents in elderly patients undergoing gastrointestinal surgery (12.9% and 11.3% in the remimazolam groups vs. 6.5% in the propofol group; *P* = 0.467). A recent large-scale non-inferiority trial involving 432 elderly patients undergoing gastrectomy reported a POD incidence of 7.9% in both the remimazolam and propofol groups (22), closely aligning with the 6.5% incidence found in our propofol group. These findings are consistent with a recent meta-analysis of five RCTs encompassing 1,368 patients, which found no significant difference in POD risk between the remimazolam and propofol groups (RR = 0.88, 95% CI: 0.58–1.33; *P* = 0.53) (23). Notably, our study adopted a strictly standardized postoperative analgesia protocol, which eliminated opioid exposure as a confounding variable.

The hemodynamic advantages of remimazolam, including lower rates of post-induction hypotension, reduced vasopressor requirements, and fewer bradycardic events, are consistent with findings from meta-analytic evidence (24). A recent meta-analysis demonstrated that remimazolam significantly reduced the incidence of post-induction hypotension (RR = 0.50; 95% CI, 0.36–0.71; *P* < 0.001) and intraoperative hypotension (RR = 0.65; 95% CI, 0.46–0.92; *P* = 0.016)compared with propofol in elderly patients (25). Salameh et al. recently showed that remimazolam was associated with a statistically significant lower risk of intraoperative study-defined hypotensive events (RR 0.50, 95%CI 0.31–0.80) in adult cardiac surgery. Despite differences in surgical populations, their results supported our finding of superior hemodynamic outcomes with remimazolam-based anesthesia (26). These findings support the clinical suitability of remimazolam for elderly patients undergoing abdominal surgery. Both induction doses (0.15 mg/kg and 0.3 mg/kg) achieved a favorable balance between effective sedation and peri-induction hemodynamic outcomes,providing practical guidance for clinical practice.

Consistent with previous reports (17, 19, 27), remimazolam was associated with a longer time to loss of consciousness, extended extubation time, and a prolonged PACU stay. The absence of flumazenil administration in our protocol, while allowing for the assessment of unassisted recovery, might contribute to these differences and should be considered when comparing results across studies. However, remimazolam demonstrated superior injection tolerability, with no reported injection pain versus 22.6% in the propofol group. Postoperative outcomes, including PONV and emergence agitation, were comparable among the groups.

Several limitations of this study warrant consideration. First, and most importantly, the sample size calculation was based on an anticipated POD incidence of 33% in the propofol group, derived from historical data (12). However, the observed incidence was significantly lower (6.5%), which is consistent with contemporary studies reporting rates of 7%–8% in similar populations (22). This discrepancy resulted in a significant reduction in statistical power. A *post-hoc* power analysis indicated that, given the observed event rate and a sample size of 62 patients per group, the study had approximately 38% power to detect a 50% relative reduction in POD incidence (from 6.5% to 3.25%) at a two-sided alpha level of 0.05. Consequently, the study was underpowered to detect a statistically significant difference in the primary outcome, even if a clinically meaningful effect existed. Therefore, the negative finding regarding POD should be interpreted with caution and does not preclude the possibility of a modest, yet clinically relevant, effect. These findings highlight the need for larger, multicenter trials with more accurate and contemporary estimates of POD incidence.

Second, the single-center design might limit the generalizability of the findings. Third, patients with preexisting cognitive impairment were excluded, limiting the applicability of the results to high-risk populations. Fourth, detailed fluid administration data were not collected as study endpoints. Fifth, twice-daily delirium assessments may have missed transient episodes, although this uniform approach is unlikely to bias comparative analyses. Sixth, our threshold-based definition of post-induction hypotension may overestimate episodes of genuine clinical harm. Furthermore, the absence of a universal definition for post-induction hypotension in perioperative research limits cross-study comparisons of absolute hypotension incidence. Finally, hemodynamic analyses relied on discrete measurements rather than continuous waveform data, precluding the calculation of time-weighted hypotension metrics.

Despite these limitations, this study possesses notable strengths, including its prospective, randomized, and blinded design; the robust standardization of postoperative analgesic and antiemetic regimens; and the direct comparison of two remimazolam dosing strategies with propofol in a homogeneous elderly cohort. The finding that both 0.15 mg/kg and 0.3 mg/kg induction doses of remimazolam provide effective sedation with superior hemodynamic outcomes constitutes clinically actionable information for anesthesiologists treating this vulnerable population. Moreover, when interpreted in the context of recent large-scale trials reporting comparable POD outcomes (22), these results contribute to the growing body of evidence that remimazolam is a hemodynamically advantageous alternative to propofol without an apparent increase in delirium risk in elderly patients.

## Conclusions

In elderly patients undergoing laparoscopic gastrointestinal surgery, remimazolam did not demonstrate superiority over propofol in reducing the incidence of postoperative delirium (POD). Given that the observed POD rate was substantially lower than expected, this finding should be interpreted cautiously. However, remimazolam was associated with favorable peri-induction hemodynamic findings. Among the regimens evaluated, both remimazolam induction doses (0.15 mg/kg and 0.3 mg/kg) demonstrated a favorable hemodynamic safety profile, as evidenced by higher post-induction blood pressure, a lower incidence of hypotension, reduced vasopressor requirements, and fewer bradycardic events. These findings support its consideration as a safer anesthetic alternative for this vulnerable elderly population.

## Data Availability

The original contributions presented in the study are included in the article/Supplementary Material, further inquiries can be directed to the corresponding authors.
