# Supplementary material for: Remimazolam versus propofol for elderly patients undergoing laparoscopic gastrointestinal surgery: a randomized trial concentrating on delirium and hemodynamic stability
Source: Front Med (Lausanne). 2026 Oct 16;13:1992969. doi: 10.3389/fmed.2026.1992969 (PMC13719584; doi:10.3389/fmed.2026.1992969)
Supplement: Supplementary file 1 [file Supplementaryfile1.pdf]

Supplementary Table 1 . Mini-Mental State Examination (MMSE)

Patient Information

Name: ____

Gender: □ Male □ Female

Age: ____________

Education Level: □ Illiterate □ Primary School □ Secondary School □ College/University

Medical Record No.: ____

Assessment Date: ____

Past Medical History: ____

Assessor (Physician): ____

Assessment Items

I. Orientation (10 points)

What is the year? ____________ (1 point)

What is the season? ____________ (1 point)

What is the month? ____________ (1 point)

What is the date today? ____________ (1 point)

What is the day of the week? ____________ (1 point)

Which country are we in? ____________ (1 point)

Which state/city are we in? ____________ (1 point)

Which district/street/community are we in? ____________ (1 point)

Where are we now? (e.g., hospital, clinic) ____________ (1 point)

Which floor are we on? ____________ (1 point)

II. Memory (3 points)

Immediate Recall:

Say three unrelated words (e.g., "apple, table, penny"). Ask the patient to repeat them.

Score: 1 point per word correctly repeated. (Total: 3 points)

III. Attention and Calculation (5 points)

Ask the patient to subtract 7 from 100 sequentially (93, 86, 79, 72, 65).

Score: 1 point per correct subtraction. (Total: 5 points)

IV. Delayed Recall (3 points)

Ask the patient to recall the three words from Section II.

Score: 1 point per word correctly recalled. (Total: 3 points)

V. Language (9 points)

Naming (2 points):

Show a watch: "What is this?" ____________ (1 point)

Show a pen: "What is this?" ____________ (1 point)

Repetition (1 point):

Ask the patient to repeat: "No ifs, ands, or buts." ____________ (1 point)

Three-Step Command (3 points):

"Take this paper in your right hand, fold it in half, and place it on the floor."

Score: 1 point per step correctly executed. (Total: 3 points)

Reading (1 point):

Show a card with "CLOSE YOUR EYES" and ask the patient to read and obey. _____________ (1 point)

Writing (1 point):

Ask the patient to write a complete sentence (e.g., "The sky is blue."). _____________ (1 point)

Drawing (1 point):

Ask the patient to copy a intersecting pentagons design

Scoring & Interpretation

Total Score (30 points):

Normal: 27–30

Cognitive Impairment: <27

Dementia Severity:

Mild: ≥21

Moderate: 10–20

Severe: ≤9

Education-Adjusted Cutoffs (Dementia Diagnosis):

Illiterate: ≤17

Primary School: ≤20
Secondary School: ≤22
College/University: ≤23

Notes for Administrators
Orientation: Allow minor errors (e.g., ±1 day for date/week).
Memory: Repeat words up to 5 times if needed. Skip delayed recall if immediate recall fails.
Attention/Calculation: Do not correct errors during serial subtraction.
Language: Ensure sentences are spontaneous (no prompts).

Adapted from
Folstein, Marshal F., et al. "'Mini-Mental State': A Practical Method for Grading the Cognitive State of Patients for the Clinician." Journal of Psychiatric Research, vol. 12, no. 3, 1975, pp. 189– 198.

Supplementary Table 2 : Riker Sedation-Agitation Scale (SAS)

Adapted from
Riker, R. R., Picard, J. T., & Fraser, G. L. (1999). Prospective evaluation of the Sedation-Agitation

Score	Term	Description
7	Dangerous Agitation	Pulls at endotracheal tube, tries to remove catheters, climbs over bedrail, strikes at staff, thrashes side-to-side.
6	Very Agitated	Requires physical restraint, frequent verbal reminding of limits; bites endotracheal tube.
5	Agitated	Anxious or mildly agitated, attempts to sit up, calms to verbal instructions.
4	Calm and Cooperative	Calm, awakens easily, follows commands.
3	Sedated	Difficult to arouse but awakens to verbal stimuli or gentle shaking; follows simple commands but drifts off again.
2	Very Sedated	Arouses to physical stimuli but does not communicate or follow commands; may move spontaneously.
1	Unarousable	Minimal or no response to noxious stimuli; does not communicate or follow commands.

Scale for adult critically ill patients. Critical Care Medicine, 27(7), 1325-1329.*
DOI: 10. 1097/00003246-199907000-00022

Supplementary Table 3 . Distribution of Surgical Procedures by Group

Surgical Procedure	R1 Group (n=62)	R2 Group (n=62)	P Group (n=62)	P value
Gastric surgery	19 (30.6%)	17 (27.4%)	19 (30.6%)	0.847
Rectal surgery	25 (40.3%)	27 (43.5%)	21 (33.9%)	
Right hemicolectomy	12 (19.4%)	11 (17.7%)	12 (19.4%)	
Sigmoid colectomy	3 (4.8%)	4 (6.5%)	7 (11.3%)	
Left hemicolectomy	1 (1.6%)	2 (3.2%)	1 (1.6%)	
Transverse colectomy	2 (3.2%)	0 (0%)	1 (1.6%)	
Other procedures*	0 (0%)	1 (1.6%)[1]	1 (1.6%)[2]	

Data are presented as n (%). P value was calculated using the Chi-square test for comparison of overall surgical procedure distribution among the three groups.
*Other procedures include: 1 ileocecal resection; 2 partial small bowel resection.

Supplementary Table 4. Omnibus tests of model effects for heart rate (HR) using the generalized estimating equation (GEE) model

Effect	Wald chi-square	P value
Group (main effect)	9.083	0.011
Time (main effect)	87.194	<0.001
Group x time (interaction)	56.342	<0.001

Supplementary Table 5. Generalized estimating equation (GEE) parameter estimates for heart rate (HR)

Parameter	B	95% Wald CI		Wald chi-square	P value
(Intercept)	66.371	63.905	68.837	2783.025	<0.001
Group x time: R1 vs P, T0 vs T4	-7.145	-10.674	-3.617	15.751	<0.001
Group x time: R1 vs P, T1 vs T4	0.484	-2.622	3.589	0.093	0.760
Group x time: R1 vs P, T2 vs T4	1.435	-1.951	4.822	0.690	0.406
Group x time: R1 vs P, T3 vs T4	-0.935	-3.735	1.864	0.429	0.512
Group x time: R2 vs P, T0 vs T4	-5.726	-8.979	-2.473	11.902	0.001
Group x time: R2 vs P, T1 vs T4	1.677	-1.415	4.770	1.130	0.288
Group x time: R2 vs P, T2 vs T4	2.790	-0.728	6.308	2.417	0.120
Group x time: R2 vs P, T3 vs T4	-0.145	-3.344	3.054	0.008	0.929

Notes

B : unstandardized regression coefficient; 95% CI : 95% Wald confidence interval.

GEE models: normal distribution, identity link function. The dependent variable is heart rate (beats/min).

Interaction terms represent difference-in-differences estimates relative to T4.

Reference categories: group = P; time = T4. Reference coefficients are not reported.

Wald χ^2 for omnibus tests; df = 2 (group), 4 (time), 8 (group × time).

R1, R2, P: study groups; T0–T4: peri-induction time points. P < 0.001 reported as <0.001.

Supplementary Table 6. Omnibus tests of model effects for systolic blood pressure (SBP) using the generalized estimating equation (GEE) model

Effect/Parameter	Wald chi-square	P value
Group (main effect)	25.922	<0.001
Time (main effect)	898.853	<0.001
Group x time (interaction)	16.595	0.035

Supplementary Table 7. Generalized estimating equation (GEE) parameter estimates for systolic blood pressure (SBP)

Parameter	B	95% Wald CI		Wald chi-square	P value
Group x time: R1 vs P, T0 vs T4	-4.887	-12.047	2.273	1.790	0.181
Group x time: R1 vs P, T1 vs T4	6.016	-3.096	15.129	1.674	0.196
Group x time: R1 vs P, T2 vs T4	2.161	-4.419	8.741	0.414	0.520
Group x time: R1 vs P, T3 vs T4	1.581	-5.230	8.391	0.207	0.649
Group x time: R2 vs P, T0 vs T4	-5.258	-12.215	1.699	2.194	0.139
Group x time: R2 vs P, T1 vs T4	0.323	-7.841	8.486	0.006	0.938
Group x time: R2 vs P, T2 vs T4	1.161	-4.532	6.855	0.160	0.689
Group x time: R2 vs P, T3 vs T4	0.484	-5.939	6.907	0.022	0.883

Notes

B: unstandardized regression coefficient; 95% CI: 95% Wald confidence interval.

GEE models: normal distribution, identity link function. The dependent variable is systolic blood pressure (mmHg).

Interaction terms represent difference-in-differences estimates relative to T4.

Reference categories: group = P; time = T4. Reference coefficients are not reported.

Wald χ^2 for omnibus tests; df = 2 (group), 4 (time), 8 (group × time).

R1, R2, P : study groups; T0–T4 : peri-induction time points. P < 0.001 reported as <0.001.

Supplementary Table 8. Omnibus tests of model effects for diastolic blood pressure (DBP) using the generalized estimating equation (GEE) model

Effect	Wald chi-square	P value
Group (main effect)	23.722	<0.001
Time (main effect)	575.390	<0.001
Group x time (interaction)	28.210	<0.001

Supplementary Table 9. Generalized estimating equation (GEE) parameter estimates for diastolic blood pressure (DBP)

Parameter	B	95% Wald CI		Wald chi-square	P value
Group x time: R1 vs P, T0 vs T4	-5.565	-9.530	-1.599	7.562	0.006
Group x time: R1 vs P, T1 vs T4	-0.290	-3.584	3.004	0.030	0.863
Group x time: R1 vs P, T2 vs T4	1.871	-1.138	4.879	1.486	0.223
Group x time: R1 vs P, T3 vs T4	0.081	-2.716	2.878	0.003	0.955
Group x time: R2 vs P, T0 vs T4	-6.710	-10.337	-3.082	13.141	<0.001
Group x time: R2 vs P, T1 vs T4	-1.435	-3.948	1.077	1.254	0.263
Group x time: R2 vs P, T2 vs T4	1.371	-1.376	4.118	0.957	0.328
Group x time: R2 vs P, T3 vs T4	0.161	-3.009	3.331	0.010	0.921

Notes

B: unstandardized regression coefficient; 95% CI: 95% Wald confidence interval.

GEE models: normal distribution, identity link function. The dependent variable is diastolic blood pressure (mmHg).

Interaction terms represent difference-in-differences estimates relative to T4.

Reference categories: group = P; time = T4. Reference coefficients are not reported.

Wald χ^2 for omnibus tests; df = 2 (group), 4 (time), 8 (group × time).

R1, R2, P : study groups; T0–T4 : peri-induction time points. P < 0.001 reported as <0.001.
